# Supplementary figures and images for: Wnt-3a Induces Epigenetic Remodeling in Human Dental Pulp Stem Cells
Source: Cells. 2020 Mar 7;9(3):652. doi: 10.3390/cells9030652 (PMC7140622; doi:10.3390/cells9030652)

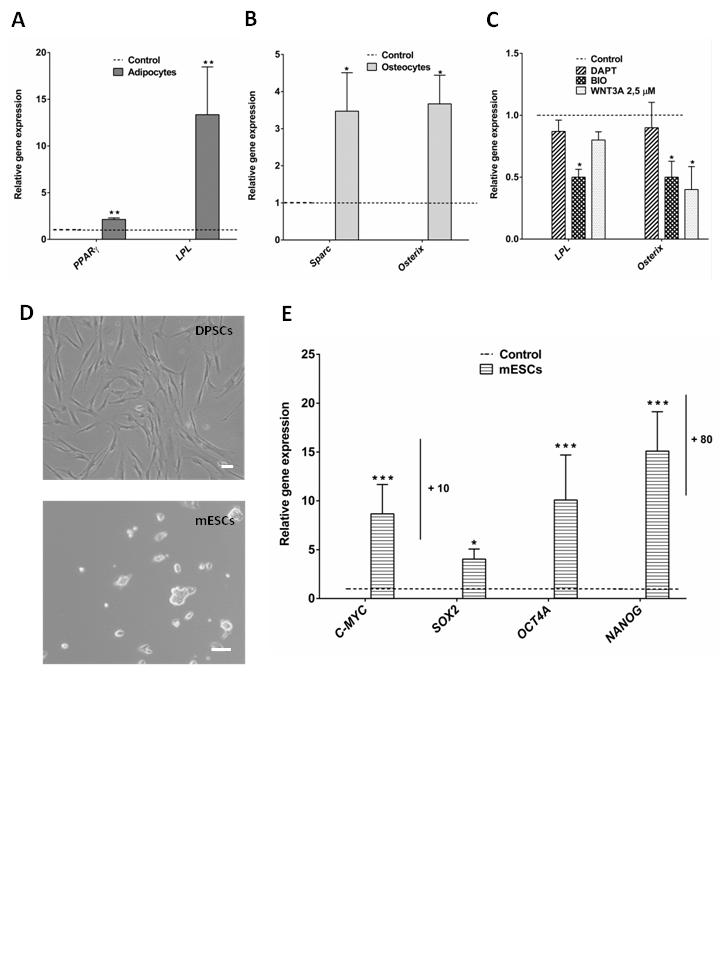

Supplement: Supplementary file 1 [file cells-09-00652-s001.zip › cells-653886-SI.tif]
